# Assessment of cerebral hemodynamics in patients with acute brain injury: a group-based multivariate trajectory approach

**DOI:** 10.3389/fneur.2026.1783194

**Published:** 2026-06-24

**Authors:** Hai Zhou, Changcun Chen, Hui Zheng, Yuheng Wu, Li Zhu, Changqing Zhou

**Affiliations:** 1 Department of Sleep Medicine, Bayi Orthopedic Hospital, China RongTong Medical Healthcare Group Co. Ltd., Chengdu, Sichuan, China; 2 Department of Neurosurgery, West China Hospital, Sichuan University, Chengdu, Sichuan, China; 3 Department of Neurosurgery, Longquan Hospital, Chengdu, Sichuan, China; 4 Department of Pain Management, Bayi Orthopedic Hospital, China RongTong Medical Healthcare Group Co. Ltd., Chengdu, Sichuan, China

**Keywords:** acute brain injury, cerebral hemodynamics, group-based multivariate trajectory, group-based trajectory modeling, longitudinal analysis

## Abstract

**Objective:**

This study aimed to describe the evolution patterns of cerebral hemodynamics in acute brain injury (ABI) patients.

**Methods:**

ABI patients with intracranial pressure (ICP), invasive arterial blood pressure, and heart rate (HR) records were identified from Medical Information Mart for the Intensive Care (MIMIC)-IV 3.0, eICU Collaborative Research Database (eICU-CRD) 2.0, and the Department of Neurosurgery of Longquan Hospital. Group-based multivariate trajectory (GBMT) modeling was employed to identify clusters of participants with similar evolution patterns of cerebral hemodynamics. Multivariate logistic regression analysis was used to examine the association between the GBMT clusters and outcomes. Features were selected using a random forest (RF) algorithm based on recursive feature elimination (RFE), and feature importance was interpreted using SHAP values. Furthermore, subgroup analyses were performed.

**Results:**

Data from 477 eligible patients from the MIMIC-IV and eICU-CRD databases were included in the trajectory analysis. Additionally, data from 519 patients from the Department of Neurosurgery were utilized for external validation. GBMT analyses identified five clusters with distinct cerebral hemodynamics evolution patterns. Compared to Cluster 1, Cluster 5 was the only one associated with an unfavorable outcome. Sensitivity analysis indicated that the effect size and direction in different subgroups were consistent, and the results were stable.

**Conclusion:**

This study identified a unique cerebral hemodynamics evolution pattern in ABI that was associated with unfavorable outcomes. Notably, our study suggested that the longitudinal analysis of multiple hemodynamic indicators may offer a novel perspective for the treatment of ABI patients.

## Introduction

Acute brain injury (ABI), including pathologies such as traumatic brain injury (TBI), intracerebral hemorrhage (ICH), and subarachnoid hemorrhage (SAH), continues to be associated with high rates of mortality and disability due to its complex pathophysiological mechanisms and substantial interindividual heterogeneity (1, 2). ABI activates complex primary and secondary brain injuries, including brain edema, brain ischemia, and intracranial hypertension (IH). In the treatment of ABI patients, intracranial pressure (ICP) and cerebral perfusion pressure (CPP) are usually considered cornerstones, with CPP calculated by subtracting ICP from mean arterial pressure (MAP) (3). However, there are still several uncertainties that need to be clarified.

The therapy driven by predetermined ICP thresholds may be misleading. The latest guidelines from the Brain Trauma Foundation (BTF) recommend the use of ICP monitoring in the management of severe traumatic brain injury (TBI). However, this recommendation was based on low-quality evidence (4). A randomized controlled trial conducted by Chesnut et al. indicated no benefit in monitoring ICP compared with imaging and clinical examination (5). The threshold of CPP also encounters the same dilemma. Consequently, longitudinal clustering statistical methods have been increasingly applied to the dynamic analysis of vital signs in ABI patients, including blood pressure, ICP, and CPP (2, 6–8).

Cerebral hemodynamics constitute an interconnected physiological system. After brain injury, impaired cardiac function may result in reduced cerebral blood flow and unfavorable functional outcomes (9). Yet few studies have performed dynamic analyses focusing on systolic blood pressure (SBP), diastolic blood pressure (DBP), and heart rate (HR) in patients with ABI. Therefore, the objective of this study was to characterize the evolution patterns of cerebral hemodynamics in ABI patients.

Whether in cross-sectional or dynamic analyses, a single indicator alone cannot reflect the prognosis of ABI patients (10). Group-based trajectory modeling (GBTM) has been widely used in longitudinal analysis (11). Recently, GBTM has been extended to multiple indicators, and this variant of the model is referred to as group-based multivariate trajectory (GBMT) (12, 13). In the era of precision medicine, bedside monitoring has generated longitudinal and multidimensional vital sign datasets. Accordingly, classification of cerebral hemodynamics phenotypes lays the foundation for subsequent investigations aimed at identifying high-risk patients and facilitating early interventions.

Thus, this exploratory study has three objectives: (1) to characterize the evolution patterns of cerebral hemodynamics in ABI patients, (2) to evaluate the relationship between these patterns and prognosis, and (3) to determine whether GBMT analysis provides additional insights beyond conventional threshold analysis.

## Methods

### Data source and ethics approvals

This study included two study cohorts: a trajectory analysis cohort and an external validation cohort. Two databases, the Medical Information Mart for Intensive Care (MIMIC)-IV version 3.0 and the eICU Collaborative Research Database (eICU-CRD) version 2.0, were utilized for the trajectory analysis. Data from the Department of Neurosurgery of Longquan Hospital were utilized for external validation.

The MIMIC-IV database is built upon MIMIC-III by integrating comprehensive, de-identified data from patients admitted to the ICUs at Beth Israel Deaconess Medical Center in Boston, Massachusetts from 2008 to 2019. This single-center database encompasses records from 383,220 unique hospital admissions (14). In contrast, the eICU-CRD is a large-scale, multi-center database that offers pre-processed data for over 200,000 ICU admissions across the United States between 2014 and 2015 (15).

Our study obtained the required license for the MIMIC-IV and eICU-CRD databases. We completed the Collaborative Institutional Training Initiative examination and obtained access rights to both databases (Certification number: 53032805). The study analyzed anonymized databases, which were pre-approved by the Institutional Review Board (IRB) at Massachusetts Institute of Technology (MIT) and Beth Israel Deaconess Medical Center (BIDMC), thus waiving the need for informed consent.

Real-world data from a local tertiary hospital’s Department of Neurosurgery were approved by the center’s institutional review board (IRB). The data spanned the period from 2009 to 2024, including physiological measurements collected by nurses via bedside monitors and documented in electronic health records. Informed consent was waived, and this research was conducted in accordance with the Helsinki Declaration.

### Study population

**Figure 1:**
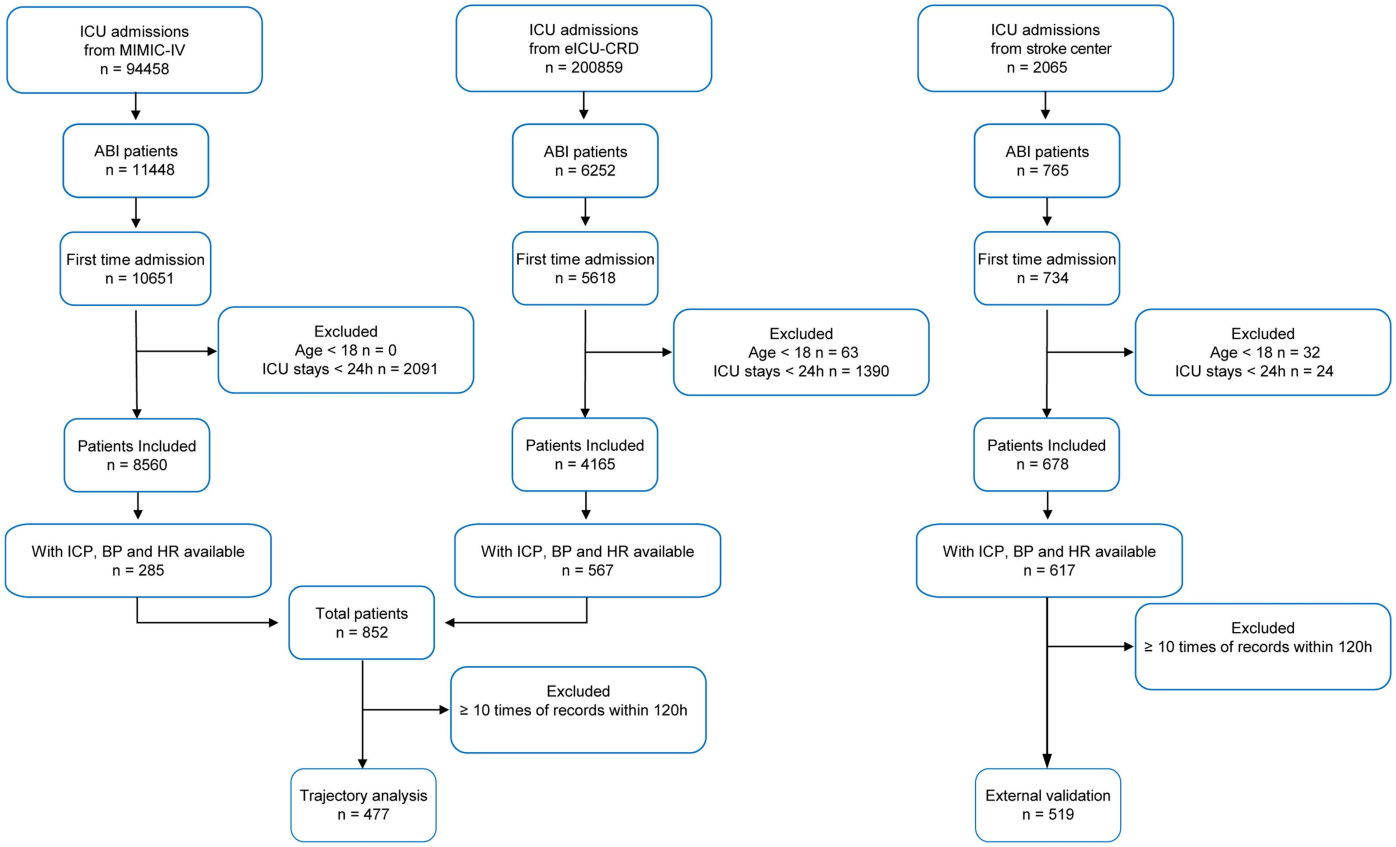
Flowchart of eligible participants in the trajectory analysis and external validation cohorts.

Patients with a diagnosis of TBI, ICH, or SAH were enrolled in the study. Individuals under the age of 18 and those who stayed in the ICU for less than 24 h were excluded. In addition, for patients who had multiple ICU admissions, only data from their first ICU admission during their initial hospitalization were included. Patients with records of ICP, invasive arterial blood pressure (ABP), and HR were ultimately enrolled in the study. The final cohort was restricted to patients with at least 10 valid measurements recorded within 120 h of ICU admission. The trajectory analysis cohort and the external validation cohort followed the same inclusion and exclusion criteria. The full study flow diagram for both cohorts is presented in Figure 1.

Detailed demographic characteristics, including age, sex, race, body mass index (BMI), comorbidities, and Charlson Comorbidity Index (CCI) derived from the recorded comorbidities, were collected. Scores of the Glasgow Coma Scale (GCS) and Sepsis-related Organ Failure Assessment (SOFA) were also extracted from MIMIC-IV and eICU-CRD. Neurosurgical interventions during the first ICU admission, including craniotomy, external ventricular drain (EVD), and cerebrospinal fluid (CSF) drainage, were collected. Other features, such as vital signs, laboratory indicators, and ICP-related parameters, were collected within 24 h after ICU admission. For variables with multiple measurements within the first 24 h of ICU admission, the most abnormal values were used.

### GBMT approach

GBTM is a semi-parametric mixture model and functions as a clustering method. The model is primarily employed to analyze longitudinal data and identify subpopulations with similar temporal evolution patterns. GBTM has been extended into the GBMT framework to accommodate multiple indicators by replacing univariate polynomial regression with multivariate regression within each group (16). This framework is termed GBMT approach (13). Based on previous studies, GBMT was applied to identify different evolution patterns of cerebral hemodynamics within 120 h after ICU admission in ABI patients, with a 4-h time interval. GBMT utilizes the expectation–maximization algorithm to perform maximum likelihood estimation from incomplete data, without relying on an explicit imputation procedure. The optimal number of groups was determined based on model fitting criteria such as Akaike information criterion (AIC), Bayesian information criterion (BIC), consistent Akaike information criterion (CAIC), sample size-adjusted Bayesian information criterion (SSBIC), and Hannan-Quinn information criterion (HQIC). The final model should have the lowest values for these model fitting criteria, and each group should have a sample size exceeding 5% of the total cohort.

### Feature selection

This study included 101 features, such as demographic characteristics, coexisting disorders, scales, neurosurgical interventions, vital signs, laboratory indicators, and ICP-related parameters. Features with missing values greater than 40% of total observations were deleted. Finally, a total of 80 features were included. The MissForest algorithm was employed to impute missing values of features (17). We employed recursive feature elimination (RFE) based on random forest (RF) to select the most relevant features, which ranks features by variable importance, iteratively eliminates the least important features, and refits the model to determine the optimal number of remaining features. The SHapley Additive exPlanations (SHAP) values were then used to visualize the importance of features selected by RF.

### Outcomes

The primary outcome was in-hospital mortality. Secondary outcomes were GCS at discharge (favorable: GCS > 8, unfavorable: GCS ≤ 8) and the difference in GCS between discharge and admission (improvement: > 0).

### Statistical analysis

Continuous variables were presented as median with interquartile range (IQR) or mean with standard deviation, while categorical variables were displayed as counts and percentages.

Continuous variables were compared using the t test (normally distributed) or Wilcoxon rank sum test (not normally distributed), while categorical variables were compared using the chi-square test or Fisher’ exact tests. Pairwise comparisons were adjusted (Bonferroni’s method). All analyses were conducted using R version 4.3.2.^1^ To ensure the reliability and reproducibility of the study, the complete source code is available via the GitHub repository.^2^

## Results

### Baseline characteristics

According to the inclusion and exclusion criteria, a total of 477 participants from the MIMIC-IV and eICU-CRD databases were finally enrolled in the trajectory analysis (Figure 1). Table 1 displays the demographic and clinical characteristics stratified by the trajectory clusters. The differences among the five clusters identified by the GBMT model were in terms of age, GCS, SOFA, CCI, the rate of ventriculostomy, and cerebral hemodynamic parameters within the first 24 h after ICU admission.

**Table 1:** Demographic and clinical characteristics stratified by trajectory clusters.

Variables	Total (*n* = 477)	Cluster 1 (*n* = 152)	Cluster 2 (*n* = 79)	Cluster 3 (*n* = 117)	Cluster 4 (*n* = 88)	Cluster 5 (*n* = 41)	*p* value
Demographics							
Age	54.0 (39.0, 66.0)	60.0 (48.0, 71.0)	46.0 (31.0, 58.5)	59.0 (50.0, 70.0)	45.0 (33.5, 58.2)	46.0 (30.0, 54.0)	<0.001
Sex							
Male	271 (56.8%)	89 (58.6%)	39 (49.4%)	60 (51.3%)	55 (62.5%)	28 (68.3%)	
Female	206 (43.2%)	63 (41.4%)	40 (50.6%)	57 (48.7%)	33 (37.5%)	13 (31.7%)	
BMI	27.0 (24.0, 31.0)	27.0 (24.0, 30.0)	27.0 (24.0, 29.5)	28.1 (25.0, 31.8)	28.0 (24.0, 31.2)	26.0 (24.0, 31.0)	0.215
Scoring systems							
GCS							
9–12	115 (24.1%)	37 (24.3%)	15 (19.0%)	47 (40.2%)	11 (12.5%)	5 (12.2%)	
≤8	362 (75.9%)	115 (75.7%)	64 (81.0%)	70 (59.8%)	77 (87.5%)	36 (87.8%)	
SOFA	8.00 (5.00, 11.0)	8.00 (5.00, 10.0)	8.00 (6.00, 12.0)	7.00 (3.00, 10.0)	9.00 (7.00, 12.0)	10.0 (7.00, 12.0)	<0.001
Charlson	1.00 (0.00, 3.00)	2.00 (0.00, 4.00)	1.00 (0.00, 2.00)	2.00 (1.00, 4.00)	0.00 (0.00, 2.00)	0.00 (0.00, 2.00)	<0.001
Surgery							
Craniotomy							
No	405 (84.9%)	126 (82.9%)	67 (84.8%)	101 (86.3%)	75 (85.2%)	36 (87.8%)	
Yes	72 (15.1%)	26 (17.1%)	12 (15.2%)	16 (13.7%)	13 (14.8%)	5 (12.2%)	
Ventriculostomy							
No	226 (47.4%)	63 (41.4%)	41 (51.9%)	46 (39.3%)	51 (58.0%)	25 (61.0%)	
Yes	251 (52.6%)	89 (58.6%)	38 (48.1%)	71 (60.7%)	37 (42.0%)	16 (39.0%)	
CSF drainage							
No	342 (71.7%)	105 (69.1%)	54 (68.4%)	84 (71.8%)	62 (70.5%)	37 (90.2%)	
Yes	135 (28.3%)	47 (30.9%)	25 (31.6%)	33 (28.2%)	26 (29.5%)	4 (9.76%)	
Day 1 indicators							
ICP	9.27 (6.53, 13.2)	9.15 (6.15, 11.7)	12.7 (7.47, 15.6)	8.46 (6.46, 10.9)	9.13 (5.97, 12.1)	14.1 (9.04, 22.1)	<0.001
SBP	130 (120, 141)	131 (122, 142)	127 (116, 145)	132 (122, 139)	128 (120, 139)	128 (118, 137)	0.732
DBP	63.7 (9.91)	61.1 (8.94)	67.1 (10.3)	64.6 (11.2)	64.7 (8.34)	62.4 (9.77)	0.001
HR	83.8 (72.7, 95.2)	78.8 (67.5, 88.3)	87.9 (77.4, 98.4)	81.3 (72.5, 87.8)	97.7 (85.2, 108)	88.5 (69.7, 100)	<0.001
MAP	86.0 (9.31)	84.8 (9.54)	87.9 (10.4)	86.6 (8.93)	86.1 (7.70)	84.6 (9.79)	0.204
PP	66.7 (17.2)	70.8 (14.9)	62.6 (22.9)	66.0 (17.6)	64.4 (14.6)	66.5 (13.2)	0.010
CPP	75.8 (69.1, 82.6)	74.7 (69.2, 81.6)	75.1 (68.3, 84.3)	78.0 (71.4, 85.5)	77.2 (69.5, 83.2)	69.5 (55.7, 75.1)	<0.001
CV of ICP	0.43 (0.30, 0.62)	0.43 (0.30, 0.63)	0.42 (0.30, 0.64)	0.48 (0.32, 0.59)	0.38 (0.32, 0.56)	0.42 (0.29, 0.66)	0.517
CV of SBP	0.10 (0.08, 0.14)	0.09 (0.07, 0.13)	0.12 (0.09, 0.16)	0.10 (0.07, 0.13)	0.09 (0.07, 0.13)	0.11 (0.09, 0.15)	0.004
CV of DBP	0.11 (0.09, 0.14)	0.10 (0.08, 0.13)	0.12 (0.09, 0.15)	0.12 (0.09, 0.15)	0.10 (0.08, 0.13)	0.11 (0.09, 0.14)	0.071
CV of HR	0.09 (0.07, 0.13)	0.09 (0.07, 0.11)	0.12 (0.09, 0.16)	0.09 (0.07, 0.12)	0.09 (0.07, 0.13)	0.09 (0.07, 0.13)	0.014
CV of MAP	0.09 (0.07, 0.12)	0.09 (0.07, 0.12)	0.10 (0.08, 0.12)	0.09 (0.08, 0.12)	0.09 (0.07, 0.11)	0.10 (0.08, 0.12)	0.219
CV of PP	0.15 (0.11, 0.22)	0.14 (0.09, 0.19)	0.19 (0.12, 0.31)	0.16 (0.11, 0.24)	0.15 (0.11, 0.21)	0.17 (0.11, 0.23)	0.004
CV of CPP	0.12 (0.09, 0.15)	0.11 (0.09, 0.14)	0.13 (0.11, 0.16)	0.11 (0.09, 0.15)	0.11 (0.09, 0.14)	0.13 (0.11, 0.23)	0.002

Data: N (%) or Mean (Q1–Q3) or mean ± standard deviation. BMI, body mass index; CCI, Charlson comorbidity index; CPP, cerebral perfusion pressure; CV, coefficient of variation; DBP, diastolic blood pressure; GCS, Glasgow coma score; HR, heart rate; ICP, intracranial pressure; MAP, mean arterial pressure; PP, Pulse pressure; SBP, systolic blood pressure; SOFA, sepsis-related organ failure assessment.

The distribution of injury types (TBI, ICH, and SAH) differed significantly across clusters (*p* < 0.001). Cluster 4 and Cluster 5 were TBI-dominant subgroups, while Cluster 1 represented an elderly subgroup with a relatively balanced distribution of the three injury types. Cluster 5 had the highest proportion of males (68.3%), the lowest median BMI [26.0 (IQR 24.0, 31.0)], the lowest GCS (87.8% of patients in this cluster had a GCS ≤ 8), and the highest median SOFA score [10.0 (IQR 7.00, 12.0)]. Meanwhile, Cluster 5 was the group with the lowest rate of surgical interventions. As for the hemodynamic indicators, Cluster 5 exhibited the highest mean ICP and relatively low SBP. Comorbidities differed among clusters mainly with respect to hypertension, cerebrovascular diseases, chronic pulmonary diseases, and diabetes (Supplementary Table S1). Supplementary Table S2 shows the comparison of baseline characteristics between the death cohort and the survival cohort in ABI patients. Among the 477 participants, 343 survived during ICU hospitalization, whereas 134 died. The survival and death cohorts exhibited significant differences primarily in age, SOFA score, CCI, mild liver disease, and the previously identified clusters. The death cohort had a significantly older median age [58.0 (IQR 46.0, 66.8) vs. 52.0 (IQR 38.0, 65.0)], a higher median SOFA score [9.00 (IQR 7.00, 12.0) vs. 8.00 (IQR 5.00, 10.0)], and a higher median CCI [2.00 (IQR 0.00, 3.75) vs. 1.00 (IQR 0.00, 3.00)]. No statistically significant difference in in-hospital mortality was observed among the three types of injury (*p* = 0.829), indicating that the overall mortality difference between clusters was not driven by the uneven distribution of underlying pathological types.

### Clusters and outcomes

**Figure 2:**
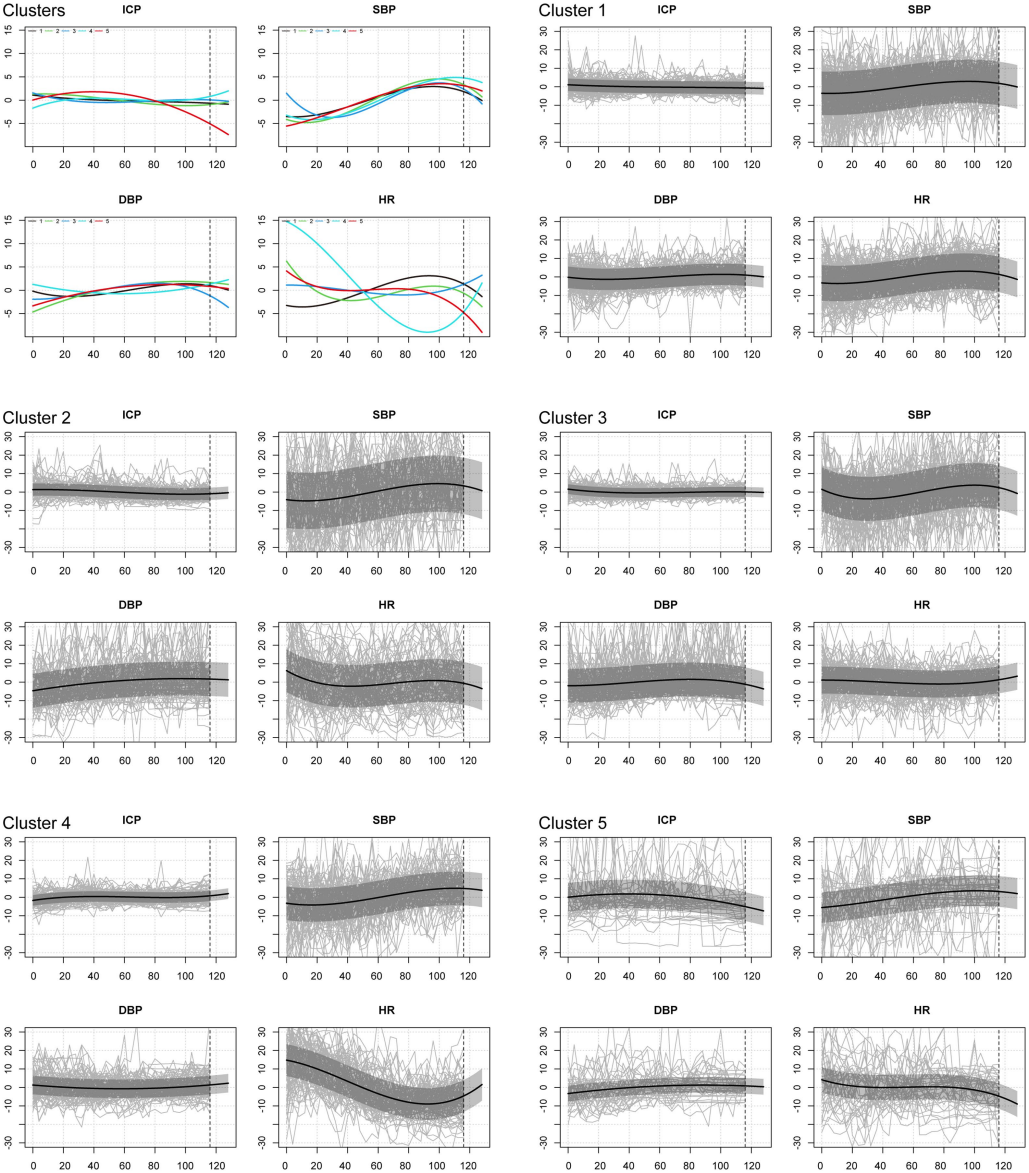
Group-based multivariate trajectory patterns of cerebral hemodynamics in the trajectory analysis cohort. The trajectories show intracranial pressure (ICP), systolic blood pressure (SBP), diastolic blood pressure (DBP), and heart rate (HR) over the first 120 hours after ICU admission across the five identified clusters.

Via a grid search algorithm, all candidate model parameters were assessed; ultimately, the 5-cluster with a 3-degree polynomial solution was identified as the optimal parameter combination (Supplementary Table S3). The parameters of the final 5-cluster 3-degree polynomial GBMT model can be found in Supplementary Table S4. Figure 2 shows five trajectory clusters of cerebral hemodynamics with their respective patterns, along with 15-min trend predictions for each cluster. Cluster 5 (*n* = 41) exhibited an evolution pattern that differed from the other clusters. This pattern was characterized by an initial rise and subsequent decline in ICP, a progressive increase in ABP, and a gradual decrease in HR.

**Figure 3:**
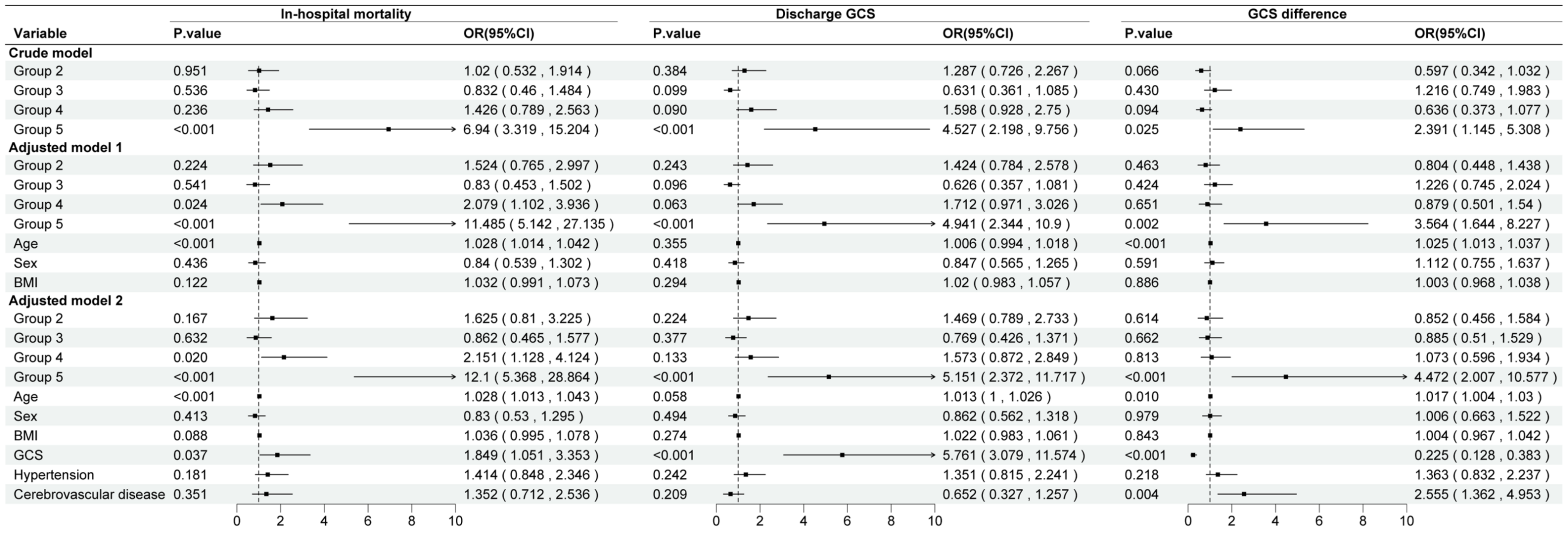
Associations between GBMT clusters and clinical outcomes in the trajectory analysis cohort. Forest plots show the odds ratios (ORs) and 95% confidence intervals (CIs) for in-hospital mortality, discharge Glasgow Coma Scale (GCS), and GCS difference in crude and adjusted logistic regression models.

Univariate and multivariate logistic regression analyses were used to analyze the relationship between clusters and outcomes. In the unadjusted models (univariate logistic regression models), compared with Cluster 1, Cluster 5 was associated with an increased risk of in-hospital mortality [odds ratio (OR) 6.692; (95% confidence interval (CI) 3.187, 14.702)], lower discharge GCS (OR 4.366; 95%CI 2.111, 9.435), and less GCS improvement (OR 2.311; 95%CI 1.103, 5.143) (Figure 3). After fully adjusting for predefined baseline confounders including age, sex, BMI, GCS, and comorbidities, multivariate logistic regression models demonstrated consistent and robust results: Cluster 5 remained significantly associated with all unfavorable outcomes, with adjusted ORs of 5.874 (95% CI 2.652, 13.008) for in-hospital mortality, 3.921 (95% CI 1.834, 8.392) for lower discharge GCS, and 2.156 (95% CI 1.002, 4.638) for reduced GCS improvement (Figure 3).

**Figure 4:**
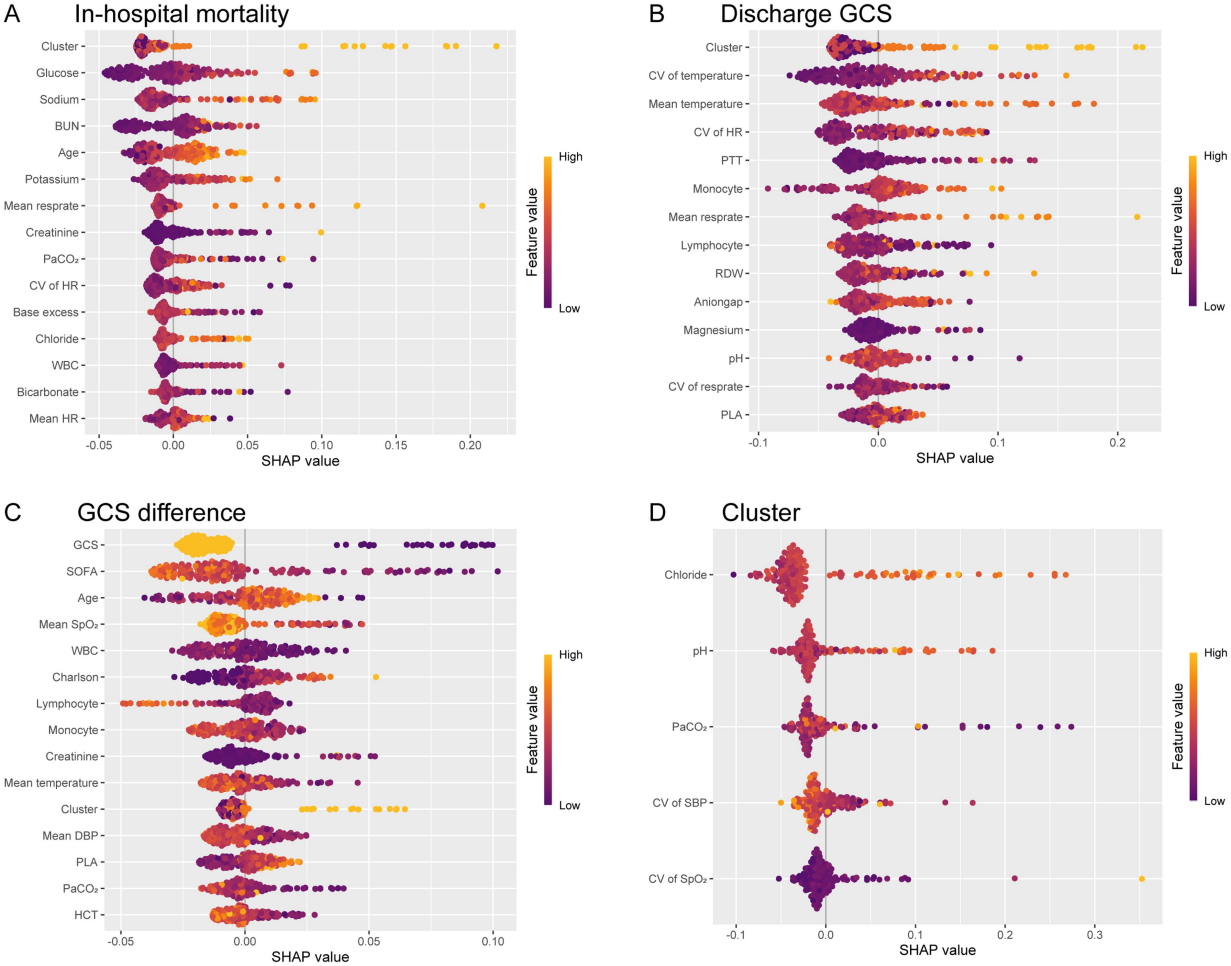
SHAP-based interpretation of predictors for unfavorable outcomes and GBMT cluster assignment. Panels A-C show feature importance for in-hospital mortality, discharge GCS, and GCS difference, respectively; panel D shows feature importance for GBMT cluster assignment.

The RF algorithm with RFE was employed to identify risk factors, and the importance of the factors was subsequently explained and visualized using SHAP values. The clusters were demonstrated to be significantly associated with unfavorable outcomes, including in-hospital mortality, discharge GCS, and difference in GCS between discharge and admission (Figures 4A–C).

### Characterization of clusters

We conducted a between-group analysis for the five clusters (Supplementary Table S5), and post-hoc pairwise comparisons were used to analyze the differences between each cluster in detail (Supplementary Table S6). Supplementary Table S5 demonstrates statistically significant differences among the five clusters in terms of the mean hemodynamic parameters and coefficient of variation for these respective indicators within 5 days. Interestingly, Cluster 5 exhibited the highest median ICP, the lowest median CPP, and the highest median CV (coefficient of variation) for both ICP and CPP. A similar situation was observed in pairwise comparisons (Supplementary Table S6), where Cluster 5 exhibited significant differences from the other clusters in terms of ICP and CPP.

Predictors of GBMT clusters were additionally analyzed, with results identifying chloride, pH, PaCO₂, CV of SBP (24 h), and CV of SpO₂ (24 h) as the most important factors for clustering outcomes (Figure 4D). The pairwise comparisons yielded different findings. Cluster 5 showed differences from the other clusters in terms of chloride and PaCO₂ on the first day. However, there were no statistically significant differences observed in the remaining predictors, including pH, CV of SBP (24 h), and CV of SpO₂ (24 h) (Supplementary Table S7).

We further evaluated inter-cluster differences in the prevalence of Cushing’s reflex (Supplementary Table S8). In the trajectory analysis cohort, the overall incidence was 0.24%, with Cluster 5 having the highest incidence (1.54%)—far higher than Cluster 1 (0.04%), Cluster 4 (0.04%), and Cluster 2 (0.51%), with no cases identified in Cluster 3. In the external validation cohort, the overall incidence was 0.09%, with Cluster 5 accounting for most cases (1.06%), only one case in Cluster 4, and no cases in Clusters 1, 2, and 3. Post-hoc pairwise comparisons confirmed that Cluster 5 had a significantly higher incidence of Cushing’s reflex than all other clusters in both cohorts (*p* < 0.001).

Clinical outcomes also displayed distinct cluster-specific disparities in both cohorts (Supplementary Table S9). In the external validation cohort, Cluster 5 comprised 41 patients, with an in-hospital mortality rate of 65.9%, which was substantially higher than the rates of 5.8, 6.45, 9.52, and 13.5% in Clusters 1–4, respectively. Consistently high mortality in Cluster 5 was also observed in the trajectory analysis cohort (68.3%). In terms of neurological outcomes, Cluster 5 had the lowest admission GCS among all clusters. Meanwhile, patients in Cluster 5 had the highest proportion of discharge GCS ≤ 8 and the highest absence of neurological functional improvement, with pairwise comparisons confirming significant differences versus all other clusters. Cluster 5 exhibited consistent phenotypic characteristics and prognostic performance across the two independent cohorts.

### Subgroup analysis

In order to validate the association between the GBMT clusters and in-hospital mortality, discharge GCS, and GCS difference, stratified analyses were performed based on factors such as age, sex, BMI, hypertension, cerebrovascular disease, diabetes, and craniotomy or ventriculostomy status. Overall, cluster 5 were linked to higher in-hospital mortality (OR 6.71, *p* < 0.001), lower discharge GCS (OR 4.37, *p* < 0.001), and reduced GCS improvement (OR 2.73, *p* = 0.006).

Interaction tests revealed no significant differences in the prognostic effect of Cluster 5 among the three types of injury (in-hospital mortality, *p* = 0.292, discharge GCS, *p* = 0.237, and GCS difference, *p* = 0.377), indicating that the trajectory-based risk stratification was generally consistent across different ABI subtypes. Subgroup analyses further demonstrated that Cluster 5 remained a consistent and significant risk factor for poor prognosis in patients with SAH and TBI, whereas no statistically significant prognostic effect was observed in patients with ICH, which may be related to the relatively small sample size of the ICH subgroup.

**Figure 5:**
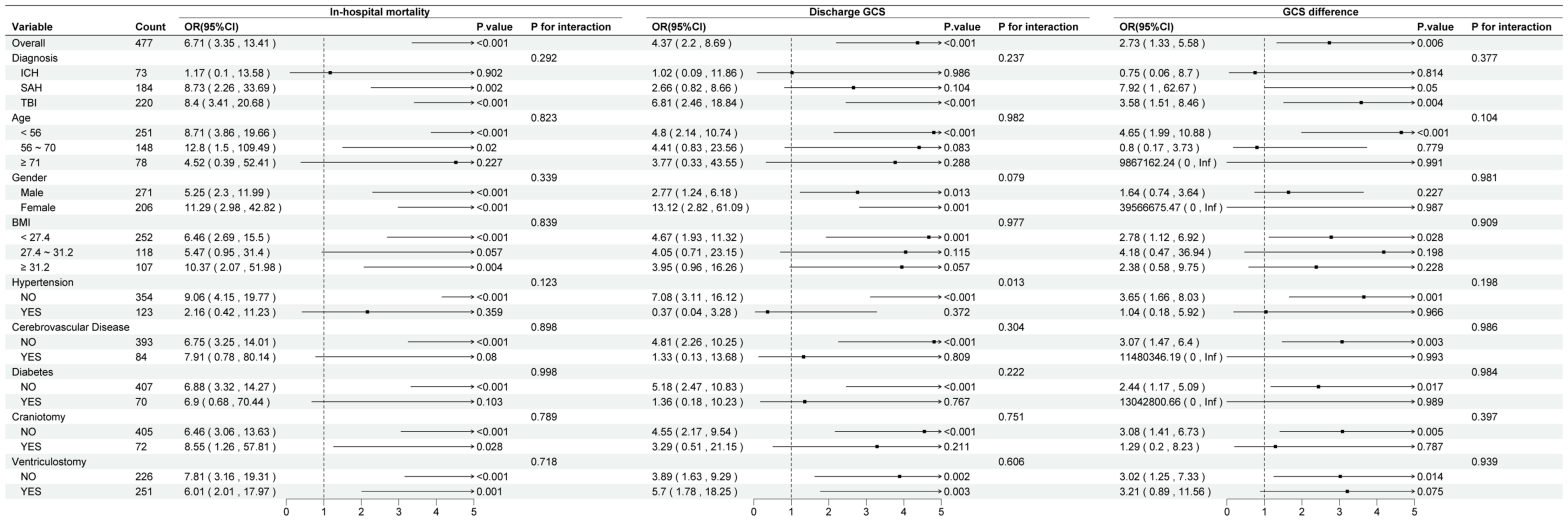
Subgroup analyses of the associations between GBMT clusters and clinical outcomes. Forest plots show ORs and 95% CIs for in-hospital mortality, discharge GCS, and GCS difference across prespecified clinical subgroups.

As shown in Figure 5, the associations between the clusters and outcomes were strongest in participants aged < 56 years and those without comorbidities (i.e., hypertension, cerebrovascular disease, and diabetes), but not statistically significant in participants aged ≥ 71 years or those with such comorbidities. A significant interaction between cluster 5 and hypertension was observed with respect to discharge GCS (*p* = 0.013): cluster 5 correlated with worse outcomes only in the non-hypertensive subgroup (OR 7.08, *p* < 0.001), not in the hypertensive subgroup (*p* = 0.372). For mortality, clusters remained significantly associated in both craniotomy and ventriculostomy subgroups. Although some OR and 95% CI estimates could not be calculated due to the relatively small sample size, the direction of the relationship between cluster 5 and outcomes remained consistent.

### External validation

**Figure 6:**
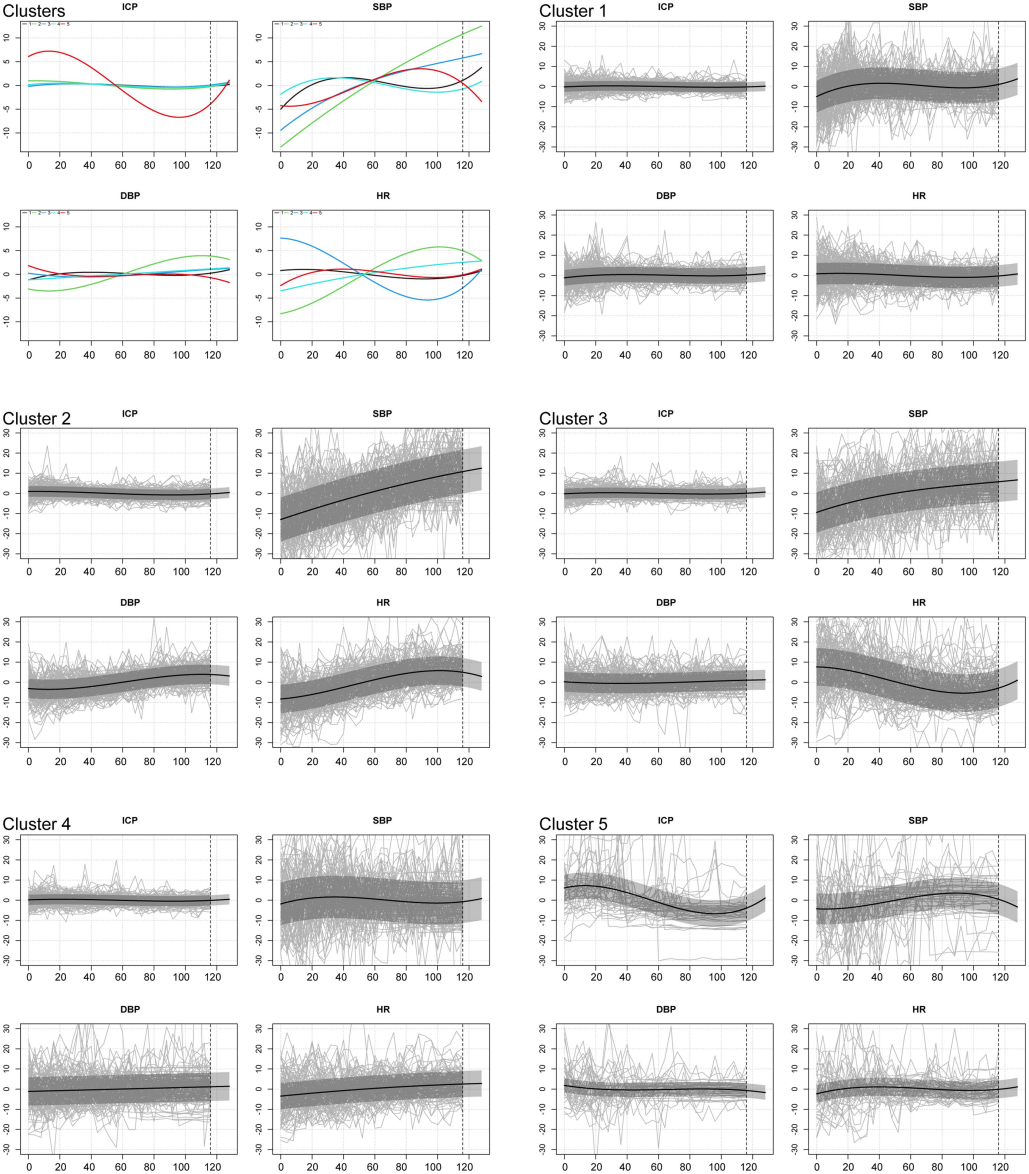
Group-based multivariate trajectory patterns of cerebral hemodynamics in the external validation cohort. The trajectories show ICP, SBP, DBP, and HR over the first 120 hours after ICU admission across the five identified clusters.

**Figure 7:**
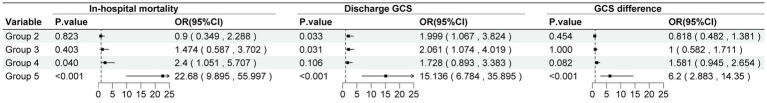
Associations between GBMT clusters and clinical outcomes in the external validation cohort. Forest plots show ORs and 95% CIs for in-hospital mortality, discharge GCS, and GCS difference.

We performed external validation by applying the identical feature processing procedure employed in the trajectory analysis cohort. A dataset of 519 participants was collected from the Department of Neurosurgery of Longquan Hospital. The same GBMT trajectory model was applied using identical parameters over the 120-h period, with measurements obtained at 4-h intervals. In the external validation cohort, Cluster 5 exhibited a slightly different evolutionary pattern of cerebral hemodynamics compared with that observed in the primary trajectory analysis cohort. This pattern was characterized by an initial rise, a subsequent decline, and a further rise in ICP, a progressive increase followed by a decline in ABP, and a gradual elevation in HR (Figure 6, Supplementary Figure S1). Univariate logistic regression analysis of this cohort yielded findings consistent with those of the trajectory analysis cohort: Cluster 5 remained significantly associated with an increased risk of in-hospital mortality (OR 22.68; 95% CI 9.895, 55.997), lower discharge GCS (OR 15.136; 95% CI 6.784, 35.895), and reduced GCS improvement (OR 6.200; 95% CI 2.883, 14.350) (Figure 7). Supplementary Table S10 summarizes the demographic and Day 1 clinical characteristics of the external validation cohort stratified by clusters, and Supplementary Table S11 lists the 5-day mean values of hemodynamic parameters. No significant inter-cluster differences were observed in demographic variables, including age, sex, and BMI. However, Cluster 5 consistently exhibited significantly higher ICP, lower CPP, and higher CV for most hemodynamic parameters, which aligned with its high-risk clinical phenotype.

## Discussion

The main findings of the study are as follows: (1) Cerebral hemodynamics evolution in ABI patients was complex and exhibited heterogeneous evolution patterns of cerebral hemodynamics; (2) Cluster 5, characterized by an initial rise and subsequent decline in ICP, a progressive increase in ABP, and a gradual decrease in HR, was independently associated with unfavorable clinical outcomes; (3) Compared with single-indicator dynamic analysis and fixed threshold-based approaches, GBMT enabled dynamic co-analysis of multiple cerebral hemodynamic indicators, thereby laying a foundation for future multivariate longitudinal studies in ABI.

Cerebrovascular hemodynamics, as a cornerstone of the management of ABI patients, has long been recognized as a critical therapeutic target. The majority of post-stroke deaths are attributed to neurological damage, whereas cardiovascular complications represent the second leading cause of post-stroke mortality (18). Research focus has shifted from fixed threshold-based approaches to dynamic analysis. In the trajectory analysis cohort, GBMT identified five distinct evolutionary patterns of cerebral hemodynamics during ICU stay. Clusters 1–4 had stable trajectories and 5-day median ICP values significantly below 20 mmHg, which were correlated with favorable outcomes. Conversely, Cluster 5 had a 5-day median ICP of 19.8 mmHg (IQR 13.0, 29.7), a value closely approaching the 22 mmHg threshold, and was associated with unfavorable outcomes (Supplementary Table S5).

Jha RM et al. previously conducted a GBTM analysis in severe TBI patients, identifying six ICP trajectory groups and including a subgroup analysis of patients who did not receive decompressive craniectomy (2). Cluster 5 in our study closely resembled Group 6 identified in their prior subgroup without decompressive craniectomy. As shown in Table 1, the majority of patients in our trajectory analysis cohort did not undergo craniotomy; this may explain why Cluster 5 in our study resembled the ICP trajectory reported in Jha RM et al.’s subgroup without decompressive craniectomy, rather than the trajectory pattern observed in their full-cohort analysis. Interestingly, Cluster 5 in our external validation cohort matched Group 6 in Jha RM et al.’s full-cohort analysis (Figure 6, Supplementary Figure S1). However, due to missing data, we could not verify the proportion of patients who had undergone decompressive craniectomy in our external validation cohort. Yang F et al. extracted data from MIMIC-IV 1.0 and eICU-CRD 1.2 databases, with only a minority of patients having undergone decompressive craniectomy. They identified six ICP trajectory groups in ABI patients, and the ICP evolution pattern of their Group 6 closely mirrored our Cluster 5 — a finding that further corroborated the reliability and generalizability of our results.

We analyzed differences in ICP spikes (defined as episodes of sustained ICP > 20 mmHg) between trajectory clusters, as shown in Supplementary Table S5. Cluster 5 had the greatest number of ICP spikes [11.0 (IQR 4.00, 19.0)], longest ICP spike duration [3.00 (IQR 1.00, 11.2)], and highest ICP spike proportion [0.22 (IQR 0.09, 0.35)], whereas Clusters 1–4 showed relatively low median values for all metrics. Jha RM et al. reported unfavorable outcomes in two trajectory groups despite overall low ICP, and they attempted but failed to explain this association through differences in cerebral compliance, neuroplasticity, or neurovascular coupling, concluding that this finding was beyond the scope of their study (2). In contrast, our findings illustrated that the frequency, duration, and proportional burden of intracranial hypertension spikes are strongly associated with trajectory clusters.

Regarding the 5-day average hemodynamic parameters, Cluster 4 exhibited the highest mean HR [87.7 (IQR 73.5, 97.0)], whereas Cluster 5 had a relatively high value [85.7 (IQR 73.0, 97.6)]. Cluster 2 displayed the highest CV of HR [0.16 (IQR 0.13, 0.21)], while Cluster 5 had a modest value [0.12 (IQR 0.10, 0.19)]. Similar patterns were observed for both SBP and DBP. Notably, Cluster 5 presented the lowest MAP [86.7 (IQR 80.6, 92.8)] and the lowest CPP [67.7 (IQR 57.7, 75.1)]. Although Cluster 5 had a modest CV of MAP, it exhibited the highest CV of CPP — a finding that suggested a bidirectional relationship between CPP variability and intense ICP fluctuations.

Neurological injury triggers excessive circulating catecholamines, which subsequently induces myocardial injury (19). Catecholamines act on the heart via *β*-adrenergic receptors, thereby increasing myocardial contractility and HR (18, 20, 21). Although initially, an elevated myocardial load has a protective effect on impaired cerebral autoregulation by maintaining cerebral blood flow, it may ultimately become maladaptive and lead to myocardial dysfunction and reduced cardiac output — a critical determinant of CPP (22). Recent observational studies (23, 24) and a meta-analysis (25) have suggested an association between *β*-blocker use and improved outcomes following TBI. A possible explanation is that *β*-blockers reduced the release of catecholamines and improved myocardial conditions (26), thereby increasing cardiac output and cerebral perfusion pressure. Furthermore, β-blockers may help maintain relative stability of cerebral hemodynamics. Patients with intracerebral hemorrhage (ICH) and non-traumatic subarachnoid hemorrhage (SAH) may have a history of hypertension and receive long-term β-blocker therapy, which may contribute to relatively favorable clinical outcomes. Additionally, the use of antihypertensive medications in response to stress-induced hypertension after neurotrauma may also be a factor affecting the cerebral hemodynamic (27). Further subgroup analyses stratified by injury type and prior β-blocker medication history are warranted to elucidate the potential impacts of these factors.

This study extended the previous trajectory analysis from a univariate to a multivariate framework and represented the first study to conduct a combined dynamic analysis of cerebral hemodynamic parameters in ABI patients. The application of GBMT in ABI patients allowed for the identification of specific evolution patterns and the outcomes associated with different patterns. The concept of “dynamic and personalized” approach has gradually replaced universal threshold values for all individuals. Future research could focus on the individualization of cerebral hemodynamic indicators and explore the combination of these indicators (e.g., cerebral vascular pressure reactivity, brain tissue oxygen tension) and clinical factors (e.g., hypostatic pneumonia, intracranial infections) to thoroughly assess and accurately predict patient outcomes. Additionally, this study was based on longitudinal cohort data from multiple centers in the United States, with all data collected from the records of bedside monitoring devices. The study incorporated high-resolution data, thereby ensured the accuracy, reliability, and stability of the results.

The study still had some limitations and required further discussion. Firstly, missing values and data noise were inevitable, and therefore, we employed the MissForest algorithm for missing value imputation. Secondly, due to the limitations of de-identified databases, detailed information on in-hospital patient management was insufficiently available, including information on prescribed medications (such as *β*-blockers and vasodilators), cardiac function data (such as electrocardiograms and echocardiograms), and neuroimages (such as CT scans and cerebral angiograms). Lastly, due to the unavailability of the 6-month Glasgow Outcome Scale (GOS) scores, GCS at discharge was a suboptimal but necessary surrogate outcome.

## Conclusion

Our findings are novel and demonstrated that multivariate longitudinal analysis of cerebral hemodynamics is essential for the comprehensive assessment of ABI patients. Cerebral hemodynamic evolution patterns following ABI clustered into five distinct subtypes, among which a potentially fatal subtype (Cluster 5) was identified. If it is rigorously validated in future studies and integrated with additional clinical indicators and high-resolution hemodynamic data, GBMT trajectory modeling could enable more refined risk stratification of ABI patients.

## Data Availability

Publicly available datasets were analyzed in this study. Data of the trajectory analysis cohort are available from the MIMIC-IV and eICU-CRD databases, which require authorized access via PhysioNet (https://physionet.org). The data extraction code for these two databases and the dataset from the external validation cohort are publicly available in the GitHub repository (https://github.com/Nicholas2074/1002-data).

## Footnotes

1
http://www.R-project.org

2
https://github.com/Nicholas2074/1002-stat

## Glossary

ABI: Acute brain injury
AIC: Akaike information criterion
APTT: Activated Partial Thromboplastin Time
AvePP: Average posterior probability
BIC: Bayesian information criterion
BMI: Body mass index
CAIC: Consistent Akaike information criterion
CCI: Charlson comorbidity index
CPP: Cerebral perfusion pressure
CV: Coefficient of variation
eICU-CRD: eICU Collaborative Research Database
GBMT: Group-based multivariate trajectory
GBTM: Group-based trajectory modeling
GCS: Glasgow coma scale
HQIC: Hannan-Quinn information criterion
ICP: Intracranial pressure
ICU: Intensive care unit
MCH: Mean Corpuscular Hemoglobin
MIMIC-IV: Medical Information Mart for the Intensive Care - IV
Occ: Odds of correct classification
PT: Prothrombin time
SHAP: Shapley Additive exPlanations
SOFA: Sepsis-related organ failure assessment
SSBIC: Sample size-adjusted Bayesian information criterion
