# Supplementary material for: Assessment of cerebral hemodynamics in patients with acute brain injury: a group-based multivariate trajectory approach
Source: Front Neurol. 2026 Jun 24;17:1783194. doi: 10.3389/fneur.2026.1783194 (PMC13341458; doi:10.3389/fneur.2026.1783194)
Supplement: Supplementary file 1 [file Supplementary_file_1.docx]

**Supplementary materials**

**Table S1 Comorbidity stratified by trajectory clusters**

**Table S2 Demographic and clinical characteristics stratified by discharge survival status**

**Table S3 The selection process of multivariate trajectory model**

**Table S4 The parameters of final 5-cluster solution of multivariate trajectory model**

**Table S5 Mean values of indicators over 5 days stratified by trajectory clusters in the trajectory analysis cohort**

**Table S6 Differences in vital sign indicators between trajectory clusters during 5 days of ICU admission**

**Table S7 Differences in predictors between trajectory clusters within 24 hours of ICU admission**

**Table S8 A-D: Differences in the frequency of Cushing's reflex between trajectory clusters**

**Table S9 A-D: Differences in the clinical outcomes between trajectory clusters**

**Table S10 Demographic and clinical characteristics stratified by trajectory clusters in the external validation cohort**

**Table S11 Mean values of indicators over 5 days stratified by trajectory clusters in the external validation cohort**

**Table S1 Comorbidities stratified by trajectory clusters**

| **Variables** | **Total (n = 477)** | **Cluster 1 (n = 152)** | **Cluster 2 (n = 79)** | **Cluster 3 (n = 117)** | **Cluster 4 (n = 88)** | **Cluster 5 (n = 41)** | ***p* Value** |
| --- | --- | --- | --- | --- | --- | --- | --- |
| Hypertension |  |  |  |  |  |  | 0.006 |
| No | 354(74.2%) | 104(68.4%) | 66(83.5%) | 78(66.7%) | 71(80.7%) | 35(85.4%) |  |
| Yes | 123(25.8%) | 48(31.6%) | 13(16.5%) | 39(33.3%) | 17(19.3%) | 6(14.6%) |  |
| Cerebrovascular disease |  |  |  |  |  |  | <0.001 |
| No | 393(82.4%) | 121(79.6%) | 71(89.9%) | 78(66.7%) | 86(97.7%) | 37(90.2%) |  |
| Yes | 84(17.6%) | 31(20.4%) | 8(10.1%) | 39(33.3%) | 2(2.27%) | 4(9.76%) |  |
| Paraplegia |  |  |  |  |  |  | 0.187 |
| No | 459(96.2%) | 145(95.4%) | 78(98.7%) | 109(93.2%) | 87(98.9%) | 40(97.6%) |  |
| Yes | 18(3.77%) | 7(4.61%) | 1(1.27%) | 8(6.84%) | 1(1.14%) | 1(2.44%) |  |
| Dementia |  |  |  |  |  |  | 0.620 |
| No | 475(99.6%) | 150(98.7%) | 79(100%) | 117(100%) | 88(100%) | 41(100%) |  |
| Yes | 2(0.42%) | 2(1.32%) | 0(0.00%) | 0(0.00%) | 0(0.00%) | 0(0.00%) |  |
| Myocardial infarct |  |  |  |  |  |  | 0.492 |
| No | 469(98.3%) | 149(98.0%) | 79(100%) | 113(96.6%) | 87(98.9%) | 41(100%) |  |
| Yes | 8(1.68%) | 3(1.97%) | 0(0.00%) | 4(3.42%) | 1(1.14%) | 0(0.00%) |  |
| Congestive heart failure |  |  |  |  |  |  | 0.209 |
| No | 469(98.3%) | 146(96.1%) | 79(100%) | 116(99.1%) | 87(98.9%) | 41(100%) |  |
| Yes | 8(1.68%) | 6(3.95%) | 0(0.00%) | 1(0.85%) | 1(1.14%) | 0(0.00%) |  |
| Peripheral vascular disease |  |  |  |  |  |  | 0.344 |
| No | 467(97.9%) | 147(96.7%) | 78(98.7%) | 113(96.6%) | 88(100%) | 41(100%) |  |
| Yes | 10(2.10%) | 5(3.29%) | 1(1.27%) | 4(3.42%) | 0(0.00%) | 0(0.00%) |  |
| Chronic pulmonary disease |  |  |  |  |  |  | 0.036 |
| No | 450(94.3%) | 143(94.1%) | 77(97.5%) | 104(88.9%) | 85(96.6%) | 41(100%) |  |
| Yes | 27(5.66%) | 9(5.92%) | 2(2.53%) | 13(11.1%) | 3(3.41%) | 0(0.00%) |  |
| Rheumatic disease |  |  |  |  |  |  | . |
| No | 473(99.2%) | 151(99.3%) | 78(98.7%) | 116(99.1%) | 87(98.9%) | 41(100%) |  |
| Yes | 4(0.84%) | 1(0.66%) | 1(1.27%) | 1(0.85%) | 1(1.14%) | 0(0.00%) |  |
| Peptic ulcer disease |  |  |  |  |  |  | 0.454 |
| No | 472(99.0%) | 151(99.3%) | 78(98.7%) | 114(97.4%) | 88(100%) | 41(100%) |  |
| Yes | 5(1.05%) | 1(0.66%) | 1(1.27%) | 3(2.56%) | 0(0.00%) | 0(0.00%) |  |
| Mild liver disease |  |  |  |  |  |  | 0.884 |
| No | 472(99.0%) | 150(98.7%) | 78(98.7%) | 115(98.3%) | 88(100%) | 41(100%) |  |
| Yes | 5(1.05%) | 2(1.32%) | 1(1.27%) | 2(1.71%) | 0(0.00%) | 0(0.00%) |  |
| Severe liver disease |  |  |  |  |  |  | 0.620 |
| No | 475(99.6%) | 150(98.7%) | 79(100%) | 117(100%) | 88(100%) | 41(100%) |  |
| Yes | 2(0.42%) | 2(1.32%) | 0(0.00%) | 0(0.00%) | 0(0.00%) | 0(0.00%) |  |
| Renal disease |  |  |  |  |  |  | 0.796 |
| No | 465(97.5%) | 147(96.7%) | 76(96.2%) | 115(98.3%) | 86(97.7%) | 41(100%) |  |
| Yes | 12(2.52%) | 5(3.29%) | 3(3.80%) | 2(1.71%) | 2(2.27%) | 0(0.00%) |  |
| Diabetes |  |  |  |  |  |  | 0.021 |
| No | 407(85.3%) | 120(78.9%) | 72(91.1%) | 97(82.9%) | 81(92.0%) | 37(90.2%) |  |
| Yes | 70(14.7%) | 32(21.1%) | 7(8.86%) | 20(17.1%) | 7(7.95%) | 4(9.76%) |  |
| Malignant cancer |  |  |  |  |  |  | 0.321 |
| No | 457(95.8%) | 144(94.7%) | 78(98.7%) | 110(94.0%) | 84(95.5%) | 41(100%) |  |
| Yes | 20(4.19%) | 8(5.26%) | 1(1.27%) | 7(5.98%) | 4(4.55%) | 0(0.00%) |  |
| Metastatic solid tumor |  |  |  |  |  |  | 0.620 |
| No | 475(99.6%) | 150(98.7%) | 79(100%) | 117(100%) | 88(100%) | 41(100%) |  |
| Yes | 2(0.42%) | 2(1.32%) | 0(0.00%) | 0(0.00%) | 0(0.00%) | 0(0.00%) |  |
| Aids |  |  |  |  |  |  |  |
| No | 477(100%) | 152(100%) | 79(100%) | 117(100%) | 88(100%) | 41(100%) | . |

**Table S2 Demographic and clinical characteristics stratified by discharge survival status**

| **Variables** | **Total (n = 477)** | **Survival (n = 343)** | **Death (n = 134)** | ***p* Value** |
| --- | --- | --- | --- | --- |
| **Demographics** |  |  |  |  |
| Age, year | 54.0(39.0, 66.0) | 52.0(38.0, 65.0) | 58.0(46.0, 66.8) | 0.021 |
| Sex |  |  |  | 0.488 |
| Male | 271(56.8%) | 191(55.7%) | 80(59.7%) |  |
| Female | 206(43.2%) | 152(44.3%) | 54(40.3%) |  |
| BMI, kg/m^2^ | 27.0(24.0, 31.0) | 27.0(24.0, 30.4) | 28.0(25.0, 32.0) | 0.141 |
| **Type of injury, n (%)** |  |  |  | 0.829 |
| ICH | 73 (15.3%) | 51 (14.9%) | 22 (16.4%) |  |
| SAH | 184 (38.6%) | 131 (38.2%) | 53 (39.6%) |  |
| TBI | 220 (46.1%) | 161 (46.9%) | 59 (44.0%) |  |
| **Scoring systems** |  |  |  |  |
| GCS |  |  |  | 0.105 |
| 9-12 | 115(24.1%) | 90(26.2%) | 25(18.7%) |  |
| ≤8 | 362(75.9%) | 253(73.8%) | 109(81.3%) |  |
| SOFA | 8.00(5.00, 11.0) | 8.00(5.00, 10.0) | 9.00(7.00, 12.0) | 0.001 |
| Charlson | 1.00(0.00, 3.00) | 1.00(0.00, 3.00) | 2.00(0.00, 3.75) | 0.030 |
| **Comorbidities** |  |  |  |  |
| Hypertension |  |  |  | 0.249 |
| No | 354(74.2%) | 260(75.8%) | 94(70.1%) |  |
| Yes | 123(25.8%) | 83(24.2%) | 40(29.9%) |  |
| Cerebrovascular disease |  |  |  | 0.809 |
| No | 393(82.4%) | 284(82.8%) | 109(81.3%) |  |
| Yes | 84(17.6%) | 59(17.2%) | 25(18.7%) |  |
| Paraplegia |  |  |  | 0.191 |
| No | 459(96.2%) | 333(97.1%) | 126(94.0%) |  |
| Yes | 18(3.77%) | 10(2.92%) | 8(5.97%) |  |
| Dementia |  |  |  | 0.483 |
| No | 475(99.6%) | 342(99.7%) | 133(99.3%) |  |
| Yes | 2(0.42%) | 1(0.29%) | 1(0.75%) |  |
| Myocardial infarct |  |  |  | 0.452 |
| No | 469(98.3%) | 336(98.0%) | 133(99.3%) |  |
| Yes | 8(1.68%) | 7(2.04%) | 1(0.75%) |  |
| Congestive heart failure |  |  |  | 0.452 |
| No | 469(98.3%) | 336(98.0%) | 133(99.3%) |  |
| Yes | 8(1.68%) | 7(2.04%) | 1(0.75%) |  |
| Peripheral vascular disease |  |  |  | 0.296 |
| No | 467(97.9%) | 334(97.4%) | 133(99.3%) |  |
| Yes | 10(2.10%) | 9(2.62%) | 1(0.75%) |  |
| Chronic pulmonary disease |  |  |  | 0.399 |
| No | 450(94.3%) | 326(95.0%) | 124(92.5%) |  |
| Yes | 27(5.66%) | 17(4.96%) | 10(7.46%) |  |
| Rheumatic disease |  |  |  | 0.315 |
| No | 473(99.2%) | 341(99.4%) | 132(98.5%) |  |
| Yes | 4(0.84%) | 2(0.58%) | 2(1.49%) |  |
| Peptic ulcer disease |  |  |  | 0.328 |
| No | 472(99.0%) | 338(98.5%) | 134(100%) |  |
| Yes | 5(1.05%) | 5(1.46%) | 0(0.00%) |  |
| Mild liver disease |  |  |  | 0.024 |
| No | 472(99.0%) | 342(99.7%) | 130(97.0%) |  |
| Yes | 5(1.05%) | 1(0.29%) | 4(2.99%) |  |
| Severe liver disease |  |  |  | . |
| No | 475(99.6%) | 341(99.4%) | 134(100%) |  |
| Yes | 2(0.42%) | 2(0.58%) | 0(0.00%) |  |
| Renal disease |  |  |  | 0.105 |
| No | 465(97.5%) | 337(98.3%) | 128(95.5%) |  |
| Yes | 12(2.52%) | 6(1.75%) | 6(4.48%) |  |
| Diabetes |  |  |  | 0.414 |
| No | 407(85.3%) | 296(86.3%) | 111(82.8%) |  |
| Yes | 70(14.7%) | 47(13.7%) | 23(17.2%) |  |
| Malignant cancer |  |  |  | . |
| No | 457(95.8%) | 329(95.9%) | 128(95.5%) |  |
| Yes | 20(4.19%) | 14(4.08%) | 6(4.48%) |  |
| Metastatic solid tumor |  |  |  | 0.078 |
| No | 475(99.6%) | 343(100%) | 132(98.5%) |  |
| Yes | 2(0.42%) | 0(0.00%) | 2(1.49%) |  |
| Aids |  |  |  |  |
| No | 477(100%) | 343(100%) | 134(100%) | . |
| **Surgery** |  |  |  |  |
| Craniotomy: |  |  |  | 0.056 |
| No | 405(84.9%) | 284(82.8%) | 121(90.3%) |  |
| Yes | 72(15.1%) | 59(17.2%) | 13(9.70%) |  |
| Ventriculostomy: |  |  |  | 0.542 |
| No | 226(47.4%) | 166(48.4%) | 60(44.8%) |  |
| Yes | 251(52.6%) | 177(51.6%) | 74(55.2%) |  |
| CSF drainage: |  |  |  | 0.747 |
| No | 342(71.7%) | 244(71.1%) | 98(73.1%) |  |
| Yes | 135(28.3%) | 99(28.9%) | 36(26.9%) |  |

Abbreviation: BMI, body mass index; CCI, Charlson comorbidity index; GCS, Glasgow coma score; SOFA, sepsis-related organ failure assessment; CSF drainage, cerebrospinal fluid drainage

**Table S3 The selection process of multivariate trajectory model**

| Degree | Groups | AIC | BIC | CAIC | SSBIC | HQIC |
| --- | --- | --- | --- | --- | --- | --- |
| 2 | 2 | 398713.0 | 399053.6 | 399098.6 | 398910.6 | 398826.3 |
| 3 | 2 | 398634.4 | 399005.2 | 399054.2 | 398849.5 | 398757.7 |
| 2 | 3 | 394317.9 | 394809.8 | 394874.8 | 394603.3 | 394481.5 |
| 3 | 3 | 394208.0 | 394805.9 | 394884.9 | 394554.8 | 394406.8 |
| 2 | 4 | 392646.1 | 393312.1 | 393400.1 | 393032.5 | 392867.6 |
| 3 | 4 | 392476.3 | 393263.5 | 393367.5 | 392933.0 | 392738.1 |
| 2 | 5 | 390756.3 | 391611.6 | 391724.6 | 391252.5 | 391040.7 |
| 3 | 5 | 390571.0 | 391554.9 | 391684.9 | 391141.8 | 390898.2 |
| 2 | 6 | 390588.5 | 391617.8 | 391753.8 | 391185.6 | 390930.8 |
| 3 | 6 | 390385.8 | 391566.6 | 391722.6 | 391070.8 | 390778.5 |

Abbreviations: AIC = Akaike information criterion; BIC = Bayesian information criterion; CAIC = Consistent Akaike information criterion; SSBIC = sample size-adjusted Bayesian information criterion; HQIC = Hannan-Quinn information criterion

**Table S4 The parameters of final 5-cluster solution of multivariate trajectory model**

Cluster 1: 152 units (APPA: 0.9891)

|  | (Intercept) | I(t^1) | I(t^2) | I(t^3) |
| --- | --- | --- | --- | --- |
| ICP | 1.2511896 | -0.1690060 | 0.006963771 | -0.0001146507 |
| SBP | -3.3605180 | -0.1943178 | 0.045767600 | -0.0011171665 |
| DBP | 0.2461138 | -0.4687008 | 0.040888036 | -0.0008141238 |
| HR | -2.9111483 | -0.3877062 | 0.062440239 | -0.0014926795 |

Cluster 2: 80 units (APPA: 0.989)

|  | (Intercept) | I(t^1) | I(t^2) | I(t^3) |
| --- | --- | --- | --- | --- |
| ICP | 1.243181 | 0.08039597 | -0.01688142 | 0.0003928048 |
| SBP | -3.597917 | -0.60182484 | 0.08350793 | -0.0018581638 |
| DBP | -5.199614 | 0.55937629 | -0.01101233 | 0.0000000000 |
| HR | 8.258753 | -2.14739556 | 0.13614901 | -0.0024812699 |

Cluster 3: 117 units (APPA: 0.978)

|  | (Intercept) | I(t^1) | I(t^2) | I(t^3) |
| --- | --- | --- | --- | --- |
| ICP | 1.980403 | -0.45146375 | 0.02556906 | -0.0004219537 |
| SBP | 3.157175 | -1.81670631 | 0.14231674 | -0.0027543139 |
| DBP | -1.865236 | -0.07571836 | 0.02994198 | -0.0008876649 |
| HR | 1.094542 | 0.04774266 | -0.02012773 | 0.0006256032 |

Cluster 4: 88 units (APPA: 0.9881)

|  | (Intercept) | I(t^1) | I(t^2) | I(t^3) |
| --- | --- | --- | --- | --- |
| ICP | -2.234992 | 0.5675216 | -0.037742807 | 0.0007400183 |
| SBP | -2.677400 | -0.6195093 | 0.071148546 | -0.0014074028 |
| DBP | 1.579073 | -0.2991187 | 0.009700477 | 0.0000000000 |
| HR | 15.265317 | -0.4333970 | -0.089580198 | 0.0027311868 |

Cluster 5: 40 units (APPA: 0.9893)

|  | (Intercept) | I(t^1) | I(t^2) | I(t^3) |
| --- | --- | --- | --- | --- |
| ICP | -0.3484696 | 0.3999159 | -0.018562327 | 0.0000000000 |
| SBP | -5.8010408 | 0.2300418 | 0.022707146 | -0.0006827807 |
| DBP | -3.7590376 | 0.4314522 | -0.009291818 | 0.0000000000 |
| HR | 5.2338246 | -1.1738173 | 0.083165108 | -0.0018384358 |

**Table S5 Mean values of indicators over 5 days stratified by trajectory clusters in the trajectory analysis cohort**

| **Variables** | **Total (n = 477)** | **Cluster 1 (n = 152)** | **Cluster 2 (n = 79)** | **Cluster 3 (n = 117)** | **Cluster 4 (n = 88)** | **Cluster 5 (n = 41)** | **p Value** |
| --- | --- | --- | --- | --- | --- | --- | --- |
| ICP | 9.47(6.91, 12.5) | 8.98(6.35, 11.2) | 10.2(7.78, 14.3) | 8.64(6.45, 10.5) | 10.3(7.75, 13.4) | 19.8(13.0, 29.7) | <0.001 |
| SBP | 133(126, 145) | 135(127, 146) | 134(125, 150) | 133(125, 141) | 133(128, 143) | 132(124, 143) | 0.203 |
| DBP | 66.7(10.8) | 62.4(8.51) | 72.1(11.1) | 70.2(12.8) | 65.1(8.42) | 65.4(8.97) | <0.001 |
| HR | 82.5(73.5, 92.3) | 81.7(72.9, 92.2) | 84.2(74.1, 93.3) | 80.7(73.4, 87.6) | 87.7(73.5, 97.0) | 85.7(73.0, 97.6) | 0.032 |
| MAP | 88.7(82.9, 95.9) | 86.9(80.8, 93.1) | 94.5(85.5, 99.8) | 89.8(84.2, 99.1) | 88.7(83.7, 92.6) | 86.7(80.6, 92.8) | <0.001 |
| PP | 68.6(16.2) | 74.6(12.8) | 65.6(18.7) | 62.5(18.4) | 69.6(12.7) | 67.4(15.0) | <0.001 |
| CPP | 78.2(72.1, 85.6) | 77.8(72.2, 83.9) | 80.4(74.3, 90.0) | 81.4(74.3, 90.9) | 78.1(71.5, 82.7) | 67.7(57.7, 75.1) | <0.001 |
| CV of ICP | 0.48(0.35, 0.62) | 0.47(0.35, 0.59) | 0.53(0.36, 0.65) | 0.48(0.36, 0.61) | 0.44(0.33, 0.55) | 0.63(0.35, 0.78) | 0.020 |
| CV of SBP | 0.12(0.10, 0.15) | 0.11(0.09, 0.13) | 0.16(0.13, 0.20) | 0.13(0.11, 0.16) | 0.11(0.09, 0.13) | 0.15(0.12, 0.16) | <0.001 |
| CV of DBP | 0.14(0.11, 0.18) | 0.11(0.10, 0.13) | 0.18(0.14, 0.22) | 0.18(0.14, 0.22) | 0.11(0.10, 0.14) | 0.15(0.13, 0.17) | <0.001 |
| CV of HR | 0.13(0.10, 0.16) | 0.12(0.09, 0.15) | 0.16(0.13, 0.21) | 0.11(0.09, 0.13) | 0.14(0.11, 0.18) | 0.12(0.10, 0.19) | <0.001 |
| CV of MAP | 0.11(0.09, 0.14) | 0.10(0.08, 0.12) | 0.14(0.11, 0.17) | 0.13(0.10, 0.15) | 0.10(0.09, 0.12) | 0.12(0.10, 0.15) | <0.001 |
| CV of PP | 0.21(0.15, 0.32) | 0.16(0.12, 0.21) | 0.34(0.25, 0.42) | 0.32(0.23, 0.42) | 0.17(0.15, 0.21) | 0.21(0.16, 0.28) | <0.001 |
| CV of CPP | 0.14(0.11, 0.16) | 0.12(0.10, 0.14) | 0.16(0.14, 0.20) | 0.15(0.12, 0.17) | 0.12(0.10, 0.14) | 0.18(0.16, 0.28) | <0.001 |
| Number of ICP spikes | 7.00(3.00, 17.0) | 5.00(2.00, 10.0) | 8.00(3.00, 16.0) | 4.00(1.00, 15.0) | 9.00(4.00, 21.0) | 11.0(4.00, 19.0) | <0.001 |
| Duration of ICP spikes | 2.00(1.00, 6.00) | 2.00(1.00, 4.00) | 3.00(1.00, 7.00) | 2.00(1.00, 5.00) | 2.00(1.00, 5.00) | 3.00(1.00, 11.2) | <0.001 |
| Proportion of ICP spikes | 0.07(0.02, 0.15) | 0.03(0.01, 0.06) | 0.08(0.03, 0.12) | 0.03(0.00, 0.12) | 0.07(0.02, 0.15) | 0.22(0.09, 0.35) | <0.001 |

Data: N (%) or Mean (Q1–Q3) or mean ± standard deviation. ICP spikes: ICP > 20mmHg

**Table S6 Differences in vital sign indicators between trajectory clusters during 5 days of ICU admission**

| **Variables** | **p.1vs2** | **p.1vs3** | **p.1vs4** | **p.1vs5** | **p.2vs3** | **p.2vs4** | **p.2vs5** | **p.3vs4** | **p.3vs5** | **p.4vs5** |
| --- | --- | --- | --- | --- | --- | --- | --- | --- | --- | --- |
| Mean ICP | 0.137 | 1.000 | 0.530 | <0.001 | 0.034 | 1.000 | <0.001 | 0.147 | <0.001 | <0.001 |
| Mean SBP | 1.000 | 0.260 | 1.000 | 1.000 | 0.210 | 1.000 | 0.800 | 1.000 | 1.000 | 1.000 |
| Mean DBP | <0.001 | <0.001 | 0.477 | 0.902 | 1.000 | <0.001 | 0.007 | 0.004 | 0.099 | 1.000 |
| Mean HR | 1.000 | 1.000 | 0.331 | 0.422 | 0.315 | 1.000 | 1.000 | 0.077 | 0.135 | 1.000 |
| Mean MAP | <0.001 | 0.012 | 1.000 | 1.000 | 0.351 | 0.001 | 0.010 | 0.415 | 0.674 | 1.000 |
| Mean PP | <0.001 | <0.001 | 0.186 | 0.091 | 1.000 | 0.949 | 1.000 | 0.013 | 0.866 | 1.000 |
| Mean CPP | 0.023 | 0.018 | 1.000 | <0.001 | 1.000 | 0.027 | <0.001 | 0.025 | <0.001 | <0.001 |
| CV of ICP | 1.000 | 1.000 | 1.000 | 0.034 | 1.000 | 0.386 | 0.636 | 1.000 | 0.018 | 0.004 |
| CV of SBP | <0.001 | <0.001 | 1.000 | <0.001 | <0.001 | <0.001 | 0.101 | <0.001 | 1.000 | <0.001 |
| CV of DBP | <0.001 | <0.001 | 1.000 | 0.009 | 1.000 | <0.001 | 0.007 | <0.001 | 0.008 | 0.010 |
| CV of HR | <0.001 | 1.000 | <0.001 | 0.007 | <0.001 | 0.140 | 0.804 | <0.001 | <0.001 | 1.000 |
| CV of MAP | <0.001 | <0.001 | 1.000 | <0.001 | 0.068 | <0.001 | 0.384 | <0.001 | 1.000 | <0.001 |
| CV of PP | <0.001 | <0.001 | 1.000 | 0.005 | 1.000 | <0.001 | <0.001 | <0.001 | <0.001 | 0.043 |
| CV of CPP | <0.001 | 0.050 | 1.000 | <0.001 | 0.334 | <0.001 | <0.001 | 0.191 | <0.001 | <0.001 |
| Number of ICP spikes | <0.001 | 0.021 | <0.001 | <0.001 | 1.000 | <0.001 | <0.001 | <0.001 | <0.001 | 0.021 |
| Duration of ICP spikes | 0.029 | 1.000 | 0.539 | <0.001 | 0.121 | 1.000 | <0.001 | 1.000 | <0.001 | <0.001 |
| Proportion of ICP spikes | <0.001 | <0.001 | <0.001 | <0.001 | 0.008 | 0.192 | <0.001 | <0.001 | <0.001 | <0.001 |

P value adjustment method: Bonferroni. Pairwise comparisons using t tests with pooled SD

**Table S7 Differences in predictors between trajectory clusters within 24 hours of ICU admission**

| **Variables** | **p.1vs2** | **p.1vs3** | **p.1vs4** | **p.1vs5** | **p.2vs3** | **p.2vs4** | **p.2vs5** | **p.3vs4** | **p.3vs5** | **p.4vs5** |
| --- | --- | --- | --- | --- | --- | --- | --- | --- | --- | --- |
| Mean ICP | 0.049 | 1.000 | 1.000 | <0.001 | 0.020 | 0.289 | <0.001 | 1.000 | <0.001 | <0.001 |
| Mean SBP | 1.000 | 1.000 | 1.000 | 1.000 | 1.000 | 1.000 | 1.000 | 1.000 | 1.000 | 1.000 |
| Mean DBP | <0.001 | 0.089 | 0.126 | 1.000 | 1.000 | 1.000 | 0.168 | 1.000 | 1.000 | 1.000 |
| Mean HR | <0.001 | 1.000 | <0.001 | 0.011 | 0.090 | 0.001 | 1.000 | <0.001 | 0.296 | 0.020 |
| Mean MAP | 0.190 | 1.000 | 1.000 | 1.000 | 1.000 | 1.000 | 0.690 | 1.000 | 1.000 | 1.000 |
| Mean PP | 0.011 | 0.391 | 0.100 | 1.000 | 1.000 | 1.000 | 1.000 | 1.000 | 1.000 | 1.000 |
| Mean CPP | 1.000 | 0.890 | 1.000 | <0.001 | 1.000 | 1.000 | <0.001 | 1.000 | <0.001 | <0.001 |
| CV of ICP | 1.000 | 1.000 | 0.450 | 1.000 | 1.000 | 1.000 | 1.000 | 1.000 | 1.000 | 0.380 |
| CV of SBP | 1.000 | 1.000 | 0.450 | 1.000 | 1.000 | 1.000 | 1.000 | 1.000 | 1.000 | 0.380 |
| CV of DBP | 1.000 | 0.370 | 1.000 | 1.000 | 1.000 | 0.284 | 1.000 | 0.054 | 0.908 | 1.000 |
| CV of HR | 0.004 | 1.000 | 1.000 | 1.000 | 0.014 | 0.052 | 0.675 | 1.000 | 1.000 | 1.000 |
| CV of MAP | 1.000 | 1.000 | 1.000 | 1.000 | 1.000 | 0.410 | 1.000 | 1.000 | 1.000 | 1.000 |
| CV of PP | <0.001 | 0.052 | 1.000 | 1.000 | 1.000 | 0.016 | 0.355 | 0.386 | 1.000 | 1.000 |
| CV of CPP | 1.000 | 1.000 | 1.000 | <0.001 | 1.000 | 0.304 | 0.056 | 1.000 | <0.001 | <0.001 |
| Chloride | 0.002 | 1.000 | 0.155 | <0.001 | 0.006 | 1.000 | 0.018 | 0.285 | <0.001 | <0.001 |
| pH | 1.000 | 1.000 | 1.000 | 0.183 | 1.000 | 1.000 | 0.277 | 1.000 | 0.174 | 0.082 |
| PaCO2 | 0.910 | 1.000 | 0.009 | 0.478 | 0.592 | 1.000 | 1.000 | 0.006 | 0.323 | 1.000 |
| CV of SpO2 | 1.000 | 0.880 | 1.000 | 0.009 | 1.000 | 1.000 | 0.204 | 1.000 | 0.366 | 0.169 |

P value adjustment method: Bonferroni. Pairwise comparisons using t tests with pooled SD

**Table S8 A-D: Differences in the frequency of Cushing's reflex between trajectory clusters**

A: Frequency of Cushing's reflex stratified by trajectory clusters (trajectory analysis cohort)

| **Variables** | **Total (n = 14310)** | **Cluster 1 (n = 4560)** | **Cluster 2 (n = 2400)** | **Cluster 3(n = 3510)** | **Cluster 4 (n = 2640)** | **Cluster 5(n = 1200)** | ***p* Value** |
| --- | --- | --- | --- | --- | --- | --- | --- |
| Cushing's Reflex | 34(0.24%) | 2(0.04%) | 12(0.51%) | 0(0.00%) | 1(0.04%) | 19(1.54%) | <0.001 |

B: Frequency of Cushing's reflex is different between trajectory clusters (trajectory analysis cohort)

| **Variables** | **p.1vs2** | **p.1vs3** | **p.1vs4** | **p.1vs5** | **p.2vs3** | **p.2vs4** | **p.2vs5** | **p.3vs4** | **p.3vs5** | **p.4vs5** |
| --- | --- | --- | --- | --- | --- | --- | --- | --- | --- | --- |
| Cushing's Reflex | <0.001 | 0.565 | 1.000 | <0.001 | <0.001 | 0.004 | 0.004 | 0.537 | <0.001 | <0.001 |

C: Frequency of Cushing's reflex stratified by trajectory clusters (external validation cohort)

| **Variables** | **Total (n = 15570)** | **Cluster 1 (n = 4140)** | **Cluster 2 (n = 3720)** | **Cluster 3 (n = 3150)** | **Cluster 4 (n = 3330)** | **Cluster 5 (n = 1230)** | ***p* Value** |
| --- | --- | --- | --- | --- | --- | --- | --- |
| Cushing's Reflex | 14(0.09%) | 0(0.00%) | 0(0.00%) | 0(0.00%) | 1(0.03%) | 13(1.06%) | <0.001 |

D: Frequency of Cushing's reflex is different between trajectory clusters (external validation cohort)

| **Variables** | **p.1vs2** | **p.1vs3** | **p.1vs4** | **p.1vs5** | **p.2vs3** | **p.2vs4** | **p.2vs5** | **p.3vs4** | **p.3vs5** | **p.4vs5** |
| --- | --- | --- | --- | --- | --- | --- | --- | --- | --- | --- |
| Cushing's Reflex | . | . | 0.551 | <0.001 | . | 0.551 | <0.001 | 1.000 | <0.001 | <0.001 |

**Table S9 A-D: Differences in the clinical outcomes between trajectory clusters**

A: Clinical outcomes across different trajectory clusters (trajectory analysis cohort)

| **Variables** | **Total (n = 477)** | **Cluster 1 (n = 152)** | **Cluster 2 (n = 79)** | **Cluster 3 (n = 117)** | **Cluster 4 (n = 88)** | **Cluster 5 (n = 41)** | ***p* Value** |
| --- | --- | --- | --- | --- | --- | --- | --- |
| In-hospital mortality | 134(28.1%) | 36(23.7%) | 19(24.1%) | 24(20.5%) | 27(30.7%) | 28(68.3%) | <0.001 |
| Admission GCS | 7.00[4.00;14.0] | 9.00[5.00;15.0] | 6.00[3.00;12.5] | 12.0[6.00;15.0] | 6.00[3.00;8.25] | 4.00[3.00;10.0] | <0.001 |
| Discharge GCS | 172(36.1%) | 49(32.2%) | 30(38.0%) | 27(23.1%) | 38(43.2%) | 28(68.3%) | <0.001 |
| GCS difference | 248(52.0%) | 81(53.3%) | 32(40.5%) | 68(58.1%) | 37(42.0%) | 30(73.2%) | 0.002 |

B: Clinical outcomes are different between trajectory clusters (trajectory analysis cohort)

| **Variables** | **p.1vs2** | **p.1vs3** | **p.1vs4** | **p.1vs5** | **p.2vs3** | **p.2vs4** | **p.2vs5** | **p.3vs4** | **p.3vs5** | **p.4vs5** |
| --- | --- | --- | --- | --- | --- | --- | --- | --- | --- | --- |
| In-hospital mortality | 1.000 | 0.757 | 0.501 | <0.001 | 0.757 | 0.618 | <0.001 | 0.265 | <0.001 | <0.001 |
| Admission GCS | 0.017 | 0.130 | <0.001 | 0.002 | 0.001 | 0.259 | 0.259 | <0.001 | <0.001 | 0.706 |
| Discharge GCS | 0.520 | 0.161 | 0.161 | <0.001 | 0.061 | 0.599 | 0.009 | 0.009 | <0.001 | 0.027 |
| GCS difference | 0.147 | 0.561 | 0.160 | 0.070 | 0.070 | 0.965 | 0.010 | 0.070 | 0.160 | 0.010 |

C: Clinical outcomes across different trajectory clusters (external validation cohort)

| **Variables** | **Total (n = 519)** | **Cluster 1 (n = 138)** | **Cluster 2 (n = 124)** | **Cluster 3 (n = 105)** | **Cluster 4 (n = 111)** | **Cluster 5 (n = 41)** | ***p* Value** |
| --- | --- | --- | --- | --- | --- | --- | --- |
| In-hospital mortality | 68(13.1%) | 8(5.80%) | 8(6.45%) | 10(9.52%) | 15(13.5%) | 27(65.9%) | <0.001 |
| Admission GCS | 8.00[5.00;13.0] | 10.0[6.25;13.0] | 7.00[4.00;12.0] | 8.00[4.00;12.0] | 9.00[6.00;13.0] | 5.00[3.00;11.0] | 0.001 |
| Discharge GCS | 128(24.7%) | 19(13.8%) | 30(24.2%) | 26(24.8%) | 24(21.6%) | 29(70.7%) | <0.001 |
| GCS difference | 197(38.0%) | 46(33.3%) | 36(29.0%) | 35(33.3%) | 49(44.1%) | 31(75.6%) | <0.001 |

D: Clinical outcomes are different between trajectory clusters (external validation cohort)

| **Variables** | **p.1vs2** | **p.1vs3** | **p.1vs4** | **p.1vs5** | **p.2vs3** | **p.2vs4** | **p.2vs5** | **p.3vs4** | **p.3vs5** | **p.4vs5** |
| --- | --- | --- | --- | --- | --- | --- | --- | --- | --- | --- |
| In-hospital mortality | 1.000 | 0.563 | 0.123 | <0.001 | 0.599 | 0.183 | <0.001 | 0.599 | <0.001 | <0.001 |
| Admission GCS | 0.001 | 0.007 | 0.056 | 0.357 | 0.014 | 0.357 | 0.049 | 0.342 | 0.270 | 0.206 |
| Discharge GCS | <0.001 | 0.075 | 0.075 | 0.206 | <0.001 | 1.000 | 0.838 | <0.001 | 0.838 | <0.001 |
| GCS difference | <0.001 | 0.641 | 1.000 | 0.177 | <0.001 | 0.641 | 0.046 | <0.001 | 0.195 | <0.001 |

**Table S10 Demographic and clinical characteristics stratified by trajectory clusters in the external validation cohort**

| **Variables** | **Total (n = 519)** | **Cluster 1 (n = 138)** | **Cluster 2 (n = 124)** | **Cluster 3 (n = 105)** | **Cluster 4 (n = 111)** | **Cluster 5 (n = 41)** | ***p* Value** |
| --- | --- | --- | --- | --- | --- | --- | --- |
| **Demographics** |  |  |  |  |  |  |  |
| Age | 60.0[45.0;70.0] | 60.0[45.0;65.0] | 60.0[50.0;66.2] | 55.0[45.0;70.0] | 60.0[50.0;70.0] | 50.0[40.0;70.0] | 0.188 |
| Sex: |  |  |  |  |  |  | 0.072 |
| Male | 291(56.1%) | 76(55.1%) | 58(46.8%) | 69(65.7%) | 64(57.7%) | 24(58.5%) |  |
| Female | 228(43.9%) | 62(44.9%) | 66(53.2%) | 36(34.3%) | 47(42.3%) | 17(41.5%) |  |
| BMI | 24.8[22.9;27.7] | 24.8[23.1;27.6] | 24.5[22.5;26.3] | 25.7[22.9;29.3] | 25.7[23.1;27.7] | 24.4[22.0;26.1] | 0.096 |
| **Day 1 Indicators** |  |  |  |  |  |  |  |
| ICP | 10.4[7.57;12.9] | 9.95[6.90;11.8] | 11.1[8.60;13.7] | 9.62[5.99;12.5] | 9.49[7.18;11.7] | 18.9[15.3;23.4] | <0.001 |
| SBP | 130[120;140] | 131[126;140] | 126[116;133] | 127[115;133] | 138[129;147] | 128[120;143] | <0.001 |
| DBP | 62.3[56.4;67.5] | 63.0[58.2;67.1] | 59.2[55.4;64.0] | 64.1[58.2;67.8] | 62.8[54.9;72.2] | 64.3[59.0;74.0] | <0.001 |
| HR | 74.8[66.8;84.6] | 76.7[70.8;83.2] | 65.3[60.9;72.6] | 88.6[81.4;96.2] | 72.4[65.9;80.3] | 75.2[67.1;87.2] | <0.001 |
| MAP | 84.8[78.5;90.4] | 86.0[81.6;90.7] | 81.2[76.4;86.7] | 83.5[77.8;90.5] | 86.8[81.1;93.8] | 86.6[78.4;93.3] | <0.001 |
| PP | 67.7(13.6) | 70.2(10.2) | 65.4(10.9) | 61.5(12.6) | 74.0(16.8) | 64.8(15.6) | <0.001 |
| CPP | 74.7[68.4;80.0] | 76.3[72.2;80.7] | 69.5[63.8;76.4] | 75.0[68.5;79.3] | 77.2[71.1;84.3] | 67.4[58.6;73.8] | <0.001 |
| CV of ICP | 0.35[0.26;0.47] | 0.35[0.28;0.49] | 0.33[0.22;0.44] | 0.36[0.26;0.48] | 0.37[0.30;0.48] | 0.27[0.22;0.42] | 0.011 |
| CV of SBP | 0.11[0.09;0.13] | 0.11[0.08;0.13] | 0.12[0.09;0.14] | 0.11[0.09;0.13] | 0.12[0.09;0.14] | 0.11[0.09;0.15] | 0.060 |
| CV of DBP | 0.13[0.10;0.17] | 0.12[0.09;0.15] | 0.15[0.11;0.18] | 0.13[0.09;0.16] | 0.14[0.11;0.20] | 0.15[0.12;0.17] | 0.001 |
| CV of HR | 0.10[0.08;0.12] | 0.10[0.08;0.11] | 0.10[0.08;0.13] | 0.10[0.08;0.13] | 0.09[0.08;0.12] | 0.11[0.07;0.16] | 0.163 |
| CV of MAP | 0.12[0.09;0.14] | 0.11[0.09;0.13] | 0.12[0.10;0.15] | 0.11[0.09;0.13] | 0.12[0.10;0.15] | 0.12[0.10;0.16] | 0.004 |
| CV of PP | 0.15[0.12;0.18] | 0.14[0.11;0.17] | 0.15[0.13;0.18] | 0.17[0.13;0.19] | 0.16[0.11;0.20] | 0.16[0.13;0.20] | 0.002 |
| CV of CPP | 0.14[0.11;0.16] | 0.12[0.10;0.14] | 0.15[0.12;0.18] | 0.14[0.10;0.16] | 0.14[0.11;0.18] | 0.16[0.13;0.23] | <0.001 |

**Table S11 Mean values of indicators over 5 days stratified by trajectory clusters in the external validation cohort**

| **Variables** | **Total (n = 519)** | **Cluster 1 (n = 138)** | **Cluster 2 (n = 124)** | **Cluster 3 (n = 105)** | **Cluster 4 (n = 111)** | **Cluster 5 (n = 41)** | **p Value** |
| --- | --- | --- | --- | --- | --- | --- | --- |
| ICP | 10.3[7.64;12.7] | 9.84[7.16;11.9] | 10.3[8.23;12.2] | 9.53[7.57;12.2] | 9.27[7.32;12.0] | 21.8[14.2;28.1] | <0.001 |
| SBP | 135(14.8) | 135(12.6) | 133(15.4) | 132(14.4) | 140(14.3) | 133(19.3) | 0.001 |
| DBP | 62.9[57.6;69.0] | 63.1[58.5;67.3] | 62.0[57.0;66.5] | 63.8[58.5;70.2] | 65.0[54.5;73.9] | 65.0[58.2;72.4] | 0.109 |
| HR | 76.5[67.8;83.9] | 76.9[69.2;82.0] | 71.8[65.8;78.2] | 84.4[75.5;95.5] | 75.1[68.0;81.8] | 76.5[68.3;87.3] | <0.001 |
| MAP | 87.0[80.8;92.3] | 87.5[81.7;91.5] | 86.6[79.3;90.8] | 86.4[80.6;91.6] | 87.8[81.8;95.5] | 86.0[79.3;95.5] | 0.150 |
| PP | 71.0(13.5) | 71.8(9.91) | 71.3(11.7) | 67.1(12.5) | 74.9(16.9) | 67.1(17.3) | 0.001 |
| CPP | 76.7[70.7;82.4] | 78.0[72.5;81.2] | 75.7[70.3;81.3] | 76.7[70.3;82.6] | 79.0[72.8;86.4] | 63.1[51.2;78.8] | <0.001 |
| CV of ICP | 0.40[0.31;0.51] | 0.37[0.30;0.53] | 0.40[0.30;0.48] | 0.38[0.30;0.47] | 0.43[0.32;0.53] | 0.48[0.31;0.66] | 0.072 |
| CV of SBP | 0.12[0.10;0.14] | 0.10[0.09;0.12] | 0.12[0.11;0.15] | 0.12[0.10;0.14] | 0.12[0.10;0.14] | 0.14[0.11;0.18] | <0.001 |
| CV of DBP | 0.14[0.11;0.17] | 0.12[0.10;0.15] | 0.15[0.12;0.17] | 0.15[0.12;0.16] | 0.16[0.13;0.19] | 0.16[0.14;0.20] | <0.001 |
| CV of HR | 0.12[0.09;0.15] | 0.10[0.08;0.12] | 0.13[0.11;0.15] | 0.14[0.10;0.17] | 0.11[0.09;0.13] | 0.14[0.10;0.18] | <0.001 |
| CV of MAP | 0.12[0.10;0.14] | 0.11[0.09;0.13] | 0.13[0.11;0.15] | 0.12[0.10;0.14] | 0.12[0.10;0.15] | 0.14[0.11;0.18] | <0.001 |
| CV of PP | 0.16[0.13;0.20] | 0.14[0.11;0.16] | 0.16[0.15;0.19] | 0.19[0.15;0.22] | 0.18[0.14;0.22] | 0.20[0.15;0.28] | <0.001 |
| CV of CPP | 0.14[0.12;0.17] | 0.12[0.10;0.14] | 0.15[0.13;0.17] | 0.14[0.12;0.16] | 0.15[0.12;0.17] | 0.20[0.15;0.43] | <0.001 |
